# Clinicians can independently predict 30-day hospital readmissions as well as the LACE index

**DOI:** 10.1186/s12913-018-2833-3

**Published:** 2018-01-22

**Authors:** William Dwight Miller, Kimngan Nguyen, Sitaram Vangala, Erin Dowling

**Affiliations:** 1 Department of Pulmonary and Critical Care Medicine, University of Chicago, 5481 S. Maryland Avenue, MC6076, Chicago, IL 60637 USA; 2 David Geffen School of Medicine, University of California, Los Angeles, University of California, Los Angeles, 10833 Le Conte Ave, Los Angeles, CA 90095 USA; 3 Department of Medicine Statistics Core, University of California, Los Angeles UCLA Med-GIM & HSR, BOX 951736, 911 Broxton Ave, Los Angeles, CA 90095-1736 USA; 4 Department of Medicine, Hospitalist Services, University of California, Los Angeles, 757 Westwood Plaza, Suite 7501, Los Angeles, CA 90095 USA

**Keywords:** Quality improvement, Discharge planning, Prediction tools, Hospital readmissions, LACE index

## Abstract

**Background:**

Significant effort has been directed at developing prediction tools to identify patients at high risk of unplanned hospital readmission, but it is unclear what these tools add to clinicians’ judgment. In our study, we assess clinicians’ abilities to independently predict 30-day hospital readmissions, and we compare their abilities with a common prediction tool, the LACE index.

**Methods:**

Over a period of 50 days, we asked attendings, residents, and nurses to predict the likelihood of 30-day hospital readmission on a scale of 0–100% for 359 patients discharged from a General Medicine Service. For readmitted versus non-readmitted patients, we compared the mean and standard deviation of the clinician predictions and the LACE index. We compared receiver operating characteristic (ROC) curves for clinician predictions and for the LACE index.

**Results:**

For readmitted versus non-readmitted patients, attendings predicted a risk of 48.1% versus 31.1% (*p* < 0.001), residents predicted 45.5% versus 34.6% (p 0.002), and nurses predicted 40.2% versus 30.6% (p 0.011), respectively. The LACE index for readmitted patients was 11.3, versus 10.1 for non-readmitted patients (p 0.003). The area under the curve (AUC) derived from the ROC curves was 0.689 for attendings, 0.641 for residents, 0.628 for nurses, and 0.620 for the LACE index. Logistic regression analysis suggested that the LACE index only added predictive value to resident predictions, but not attending or nurse predictions (*p* < 0.05).

**Conclusions:**

Attendings, residents, and nurses were able to independently predict readmissions as well as the LACE index. Improvements in prediction tools are still needed to effectively predict hospital readmissions.

## Background

Thirty-day hospital readmissions are costly, and can be frustrating for both patients and clinicians. As such, they are increasingly scrutinized, and significant efforts are directed at quantifying, understanding, and preventing them.

One part of these efforts has been the development of risk models to help identify patients at risk for hospital readmission. A popular model has been the LACE index, due to its simplicity and comparable accuracy to other models [1–3]. It is calculated by taking into account the length of hospitalization (L), acuity of admission (A), the patient’s Charlson Comorbidity Index (C), and the number of visits to the emergency department during the previous 6 months (E). Scores range from 1 (representing low risk of readmission), to 19 (representing a high risk of readmission). Since its derivation, the LACE index has been found to be variably predictive of hospital readmission in published reports [4–6]. However, it continues to be utilized as a risk stratification tool due to its simplicity and the felt need for such prediction models.

The felt need for readmission prediction models derives at least in part from the belief that providers are unable to predict readmissions without them. However, the body of literature supporting this belief is thin. Allaudeen and colleagues addressed this question in *Inability of Providers to Predict Unplanned Readmissions*, where they compared the predictive ability of prospectively surveyed clinicians with the predictive ability of the Probability of Readmission score (Pra), another predictive model. Their final assessment, based on 159 patients, was that both the care providers and the Pra were equally poor predictors of readmission [7]. Their results were similar to a previous report by the ESCAPE trialists, who reported that physicians, nurses, and a separate predictions model did not successfully predict 6-month (instead of 30-day) hospital readmissions for patients with heart failure [8]. No similar comparison has been published for the LACE index, which was developed later and has been more widely adopted, and no subsequent study has directly confirmed the results that hospital care providers are poor predictors of 30-day readmissions. On the contrary, a separate study found that nurses who were surveyed about patient discharge readiness were better able to predict the discharge readiness than the patients themselves, though in this study, both emergency department visits and readmissions were combined as an outcome measure [9].

The hypothesis that providers are unable to predict hospital readmissions without the use of prediction tools is therefore supported only by a very thin body of literature. We seek to add to that body of literature with this project, in which we have designed a survey to assess providers’ ability to predict 30-day hospital readmissions, and have compared their predictive ability with that of the widely-utilized prediction tool, the LACE index.

## Methods

A 3-item survey was designed to assess the abilities of providers to predict 30-day readmissions. In the survey, we ask providers to estimate the risk of readmission on a continuous scale from 0 to 100%. We then ask them, in a second item, to indicate if the patients could be described as having any of the following risk factors for readmission: having poor understanding of their disease, having poor adherence to therapy, having poor social support or access to care, having a condition that is likely to relapse or worsen, requiring therapy that is likely to result in a complication, having a high likelihood of developing a new medical condition that would require readmission, having an organ transplant, or having a previous readmission [7, 10–12]. In the last item, the provider indicates his or her role: as a resident, attending, or nurse. The surveys were collected by a trained medical student who attempted to collect all surveys in person on the day of discharge. Surveys that could not be collected on the day of discharge were accepted within 48 h of discharge. The surveys were collected between June 4 and July 24, 2015, a period of 50 days. The LACE index was generated by our electronic health record (EHR), based on visit history and encounter diagnoses, and was collected for each patient on the day of discharge, or within 48 h of discharge. Readmissions were tracked through an electronic health system quality report derived from the health system’s EHR to identify patients discharged and readmitted from our institution, and this report was manually confirmed through chart review.

Survey respondents were the General Internal Medicine Physician attendings and residents rotating on a General Internal Medicine ward at an academic referral hospital, as well as the primary patient nurses who were supporting this service. Surveys were collected from 29 attendings, 19 residents, and 129 nurses. Attempts were made to collect surveys from each provider on each patient who was discharged from the General Internal Medicine service, resulting in 377 surveys collected. Surveys were excluded from analysis if the patients were readmitted before the surveys were completed, if they died within 30 days of their admission, if they were discharged on hospice, or if they had a POLST that specified “Do not rehospitalize”, resulting in a final sample of 359 patients for analysis.

Overall risk assessments were evaluated in two ways. First, risk scores for each provider type, as well as the LACE index, were compared between readmitted and non-readmitted patients. Scores were summarized using means and standard deviations, and were compared between groups using two-sample t-tests. Second, receiver operating characteristic (ROC) curves were estimated for each score, with discrimination evaluated using the area under the curve (AUC). Estimates and 95% confidence intervals (95% CI) for AUCs were computed for each provider type, as well as for the LACE index, and were tested for differences using logistic regression models. Additionally, value-added of risk scores relative to the LACE index was assessed using logistic regression models controlling for the patient’s LACE index. To evaluate performance of risk assessments in patients with the pre-specified risk factors for readmission, AUCs were computed for patients identified as having poor understanding of their disease, having poor adherence to therapy, having poor social support or access to care, having a condition that is likely to relapse or worsen, requiring therapy that is likely to result in a complication, having a high likelihood of developing a new medical condition that would require readmission, having an organ transplant, or having a previous readmission. No inferences were performed due to small sample sizes. We also calculated odds ratios for readmission in these pre-specified subgroups. *P*-values less than 0.05 were considered statistically significant. All analyses were performed using SAS v. 9.4 (SAS Institute Inc., Cary, NC).

Our institutional review board waived approval for this study, and all study participants provided verbal consent to participate.

## Results

For the 359 cases which we included in our analysis, 78 were readmitted to the hospital within 30 days, a readmission rate of 22%. Characteristic of the patients admitted to the General Internal Medicine Wards for the two months during which our study took place are described in Table 1.

**Table 1:** Characteristic of the patients admitted to the General Internal Medicine Wards at our hospital in June–July 2015

Patient characteristic	Percent of patients
Patient Age	
> 65	56%
< 65	44%
Patient Sex	
Male	56%
Female	44%
Primary Diagnosis by Diagnosis Related Group	
Cardiac Diagnosis (e.g. heart failure, atrial fibrillation, etc.)	27%
Infectious Diagnosis (e.g. sepsis, etc.)	18%
Respiratory Diagnosis (e.g. COPD, etc.)	7%
Malignancy-Related Diagnosis (e.g. malignant ascites, etc.)	8%
Benign Hematologic Diagnosis (e.g. sickle cell disease, etc.)	3%
Hepatopancreaticobiliary Diagnosis (e.g. pancretitis, etc.)	3%
Neurologic Diagnosis (e.g. seizures, etc.)	2%
Renal Diagnosis (e.g. AKI, etc.)	5%
Digestive Disorder (e.g. inflammatory bowel disease, etc.)	9%
Endocrine Disorder (e.g. diabetes, etc.)	2%
Musculoskeletal and Skin Diagnosis (e.g. fractures, etc.)	2%
Psychiatric Diagnosis (e.g. delirium, etc.)	3%
Other	9%

Attendings, residents, and nurses all predicted higher likelihood of readmission for the readmitted patients compared to non-readmitted patients, with differences in the predictions reaching statistical significance (*p* < 0.05, Table 2). Readmitted patients also had a mean LACE index that was significantly higher than non-readmitted patients (*p* < 0.05). Of note, there was considerable overlap in the LACE indices for readmitted and non-readmitted patients, and similar overlap for the providers’ predictions.

**Table 2:** LACE index and Clinician Subjective Predictions for Readmitted and Non-readmitted Patients

	Readmitted	Not readmitted	*P*-value
	(*N* = 78)	(*N* = 281)	
LACE Index			0.003
Mean (SD)	11.3 (3.4)	10.1 (3.3)	
Median (Q1, Q3)	12 (9, 14)	10 (8, 12)	
Min, Max	3, 18	1, 18	
Residents			0.002
Mean (SD)	45.5 (28.9)	34.6 (24.8)	
Median (Q1, Q3)	50 (20, 67.5)	30 (15, 50)	
Min, Max	0, 100	0, 100	
Missing data	6	17	
Attendings			< 0.001
Mean (SD)	48.1 (28.4)	31.1 (25.4)	
Median (Q1, Q3)	40 (25, 70)	25 (10, 50)	
Min, Max	5, 100	0, 100	
Missing data	3	7	
Nurses			0.011
Mean (SD)	40.2 (22.9)	30.6 (23.3)	
Median (Q1, Q3)	40 (25, 50)	25 (10, 50)	
Min, Max	3, 90	0, 100	
Missing data	30	87	

*SD* Standard Deviation, *Q1* 25th percentile, *Q3* 75th percentile

**Fig. 1:**
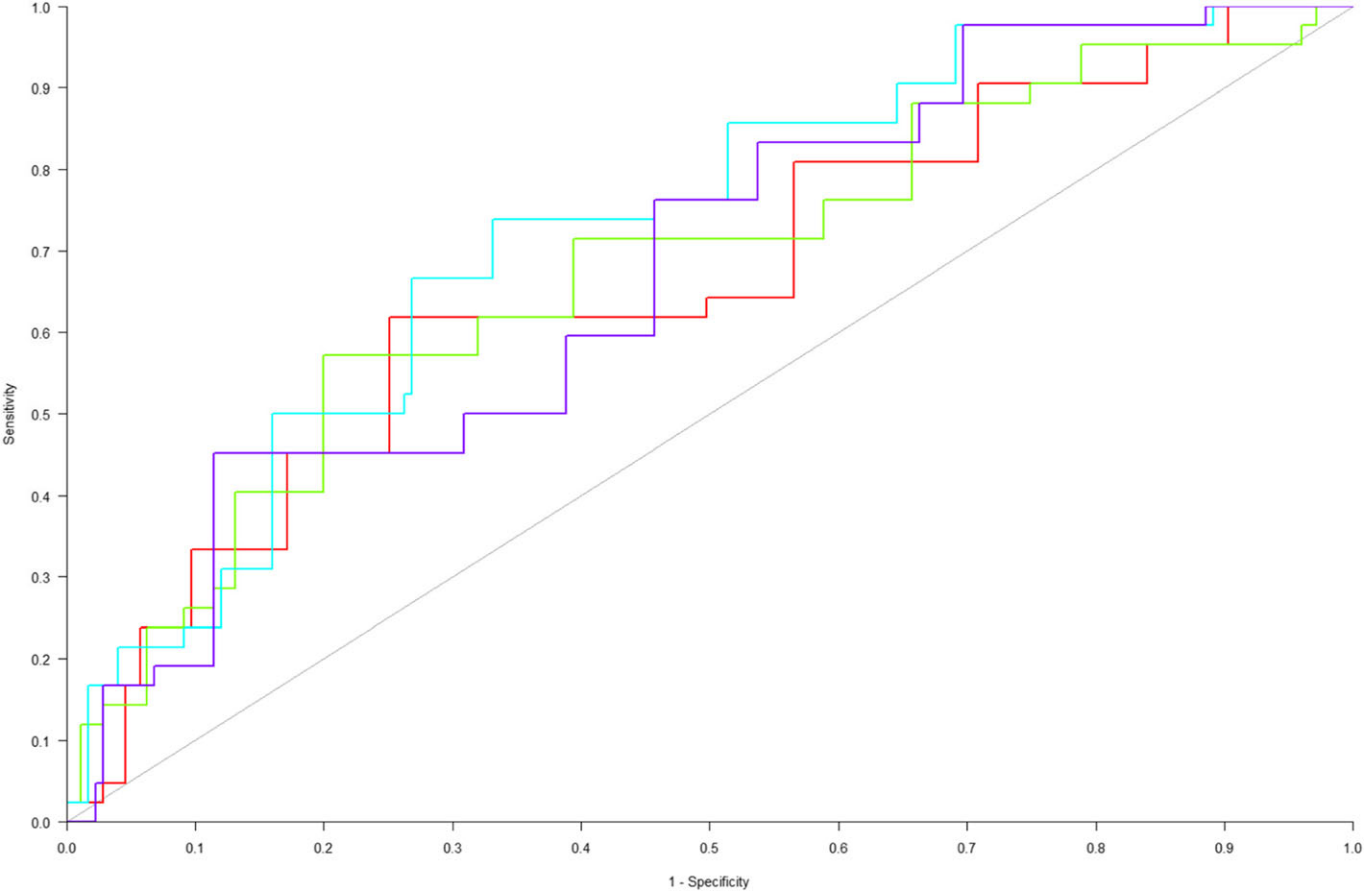
Receiver Operating Characteristic Curves for the LACE index and Clinicians’ Subjective Predictions. The figure shows the ROC curves for the LACE index (red) and for the predictions of attendings (blue), residents (green), and nurses (purple). The area under the curve (AUC) derived from the ROC curves was 0.689 for attendings (95% CI 0.603, 0.776), 0.641 for residents (95% CI 0.543, 0.739), 0.628 for nurses (95% CI 0.540, 0.716), and 0.620 for the LACE index (95% CI 0.521, 0.718)

To assess the tradeoff between the sensitivity and specificity of the LACE index and providers’ predictions, we constructed ROC curves (Fig. 1). This assessment suggested that attendings were best able to distinguish patients who would be readmitted from those who would not be, followed by residents, nurses, and the LACE index. We were not able to demonstrate statistical significance in the difference between the AUC of the different predictors and the LACE index (*p* > 0.05).

We next hypothesized that while the LACE index appeared not to be superior to the providers’ predictions in terms of distinguishing patients at risk for readmission, it might nevertheless be a useful tool if it could add additional predictive power when combined with provider predictions. However, when we assessed this using a logistic regression analysis, we found that the LACE index only added additional predictive value to residents’ predictions, but not to attendings’ or nurses’ predictions (*p* < 0.05, Table 3).

**Table 3:** Results of logistic regression analysis of LACE index added to subjective clinician predictions

Value added when LACE index added to the following provider predictions		
	OR (95% CI)	*P*
Residents	1.10 (1.02, 1.20)	0.019
Attendings	1.08 (0.99, 1.17)	0.089
Nurses	1.10 (0.99, 1.22)	0.067

Logistic regression analysis was performed to assess if adding LACE index to clinician predictions resulted in a prediction that was more likely to distinguish readmitted from non-readmitted patients. Results of this analysis were considered positive if the OR and 95% confidence intervals were greater than 1.0, with *p* < 0.05

Finally, we asked providers to subjectively identify patient characteristics that might place patients at risk for readmission. Our most notable result from this data was that nurses were able to predict 30-day hospital readmissions with an AUC of 0.778 for patients when they described them as having a poor understanding of their illness. Indeed, each provider group demonstrated areas of relative strength and weakness (Table 4), though again we were not able to demonstrate statistical significance in the differences of the providers’ predictions for different groups. When we calculated odds ratios to estimate the risk of readmission in patients identified as having the different characteristics, we found that there was an elevated risk of readmission when attendings believed that patients were poorly adherent to their therapies (OR 1.81, 95% CI 1.04, 3.16) or had severe disease (OR 2.16, 95% CI 1.21, 3.86); or when residents believed that patients were medically complex (OR 2.00, 95% CI 1.15, 3.48) or had a previous admission (OR 2.89, 95% CI 1.46, 5.72). Other subgroups of patients (e.g. those identified by nurses to have poor adherence, or those identified by attendings to have poor social support, etc) did not have an elevated risk of readmission by this analysis.

**Table 4:** AUCs for clinician predictions based on clinician-identified patient risk factors for readmission

Patient identified as having:	AUC for Clinician Predictions (95% CI)		
	Residents	Attendings	Nurses
Poor understanding	0.516 (0.355, 0.675) *n* = 79	0.713 (0.590, 0.836) *n* = 88	0.778 (0.614, 0.941) *n* = 44
Poor adherence	0.700 (0.532, 0.868) *n* = 52	0.610 (0.456, 0.765) *n* = 68	0.498 (0.280, 0.717) *n* = 40
Poor social support	0.669 (0.457, 0.881) *n* = 53	0.631 (0.432, 0.831) *n* = 52	0.593 (0.337, 0.849) *n* = 24
Severe disease	0.629 (0.542, 0.717) *n* = 206	0.649 (0.569, 0.728) *n* = 220	0.572 (0.434, 0.710) *n* = 93
High-risk therapy	0.577 (0.416, 0.738) *n* = 74	0.594 (0.461, 0.727) *n* = 87	0.616 (0.405, 0.826) *n* = 45
Medically complex	0.594 (0.464, 0.724) *n* = 88	0.601 (0.459, 0.742) *n* = 75	0.543 (0.363, 0.723) *n* = 52
Organ transplant	0.493 (0.309, 0.677) *n* = 48	0.661 (0.504, 0.818) *n* = 49	0.574 (0.314, 0.834) *n* = 30
Previous admission	0.660 (0.490, 0.829) *n* = 43	0.510 (0.266, 0.754) *n* = 26	0.547 (0.263, 0.831) *n* = 22

## Discussion

Our results suggest that attendings, residents, and nurses perform as well as the LACE index, an industry-standard and widely utilized prediction tool, in predicting 30-day hospital readmissions. The AUC for the LACE index in our population of 0.620 (95% CI 0.521, 0.718) was qualitatively consistent with the originally reported LACE index c-statistic of 0.684 (95% CI 0.679–0.691) [13]. Clinicians performed just as well, with AUCs that were higher but not statistically different from the LACE index. These results conflict with a previous report, in which neither providers nor the risk model that was assessed (the Pra) were able to outperform chance in predicting 30-day readmissions [7]. We cannot know with certainty what accounts for the difference in our results, but possibilities include the difference in our patient population, including our inclusion of clinicians’ predictions for patients over age 18 instead of only for patients over age 65; as well as the approximately 7 years that elapsed since that previous report, during which much attention and clinician effort has been directed at reducing hospital readmissions.

However, while both clinicians and the LACE index performed better than chance in predicting 30-day hospital readmissions, it is fair to question if that performance was clinically useful. Predictions based on the LACE index and clinician expertise both achieved AUCs of less than 0.7 (often considered a target for “good” predictive capacity), reflecting significant overlap in the predictions for readmitted and non-readmitted patients. In spite of the “improved” ability of physicians and a newer prediction model to predict hospital readmissions, the predictive capacity of both clinicians and the LACE index remained suboptimal.

In our patients, the limited usefulness of the LACE index appears to have been driven by overall high LACE indices for both readmitted and non-readmitted patients (Table 2). Our results are similar to previous studies in which the LACE index did not accurately predict readmissions in an older UK population, or in patients readmitted with heart failure [4, 6]. In both of these previous reports, the Charlson Comorbidity Indices (CCI) were high, resulting in a higher LACE index. Our population is that of a referral academic medical center, which also has a relatively high CCI. Our result, when considered together with the previous reports described above, has particular cautionary relevance to centers that treat patients with high CCI’s. Interestingly, the limitations in clinician predictions also seems to have been driven by an excessively high estimate of readmission risk, with clinicians predicting a higher rate of readmissions than was actually observed. These results underscore the need for ongoing efforts to improve prediction tools, as well as the need for ongoing education of clinicians.

When considering how to improve prediction tools, it will be important to focus on what these tools can add to clinician expertise. Interestingly, in our cohort, the LACE index only added predictive value to inexperienced clinicians (residents), but not to more experienced clinicians (attendings and nurses). Clearly, prediction tools should consistently offer predictive capacity not available to clinicians without them, however this criterion has generally not been assessed in the development of new prediction tools. Our results suggest that prediction tools may warrant validation in the local context of clinician expertise and patient population characteristics, if their usefulness is to be ensured, and if the significant effort and expense of implementing them in health systems is to be justified.

We and others have made initial attempts to clarify what factors play into clinician expertise (or lack thereof) regarding hospital readmissions, as this could be useful information when developing prediction tools to constructively supplement clinician expertise. We did this by asking our clinicians to identify possible risk factors for readmission, and we found that when clinicians identified patients as having poor understanding or severe disease, the odds ratio of readmission was higher; furthermore, the AUCs were highest when clinicians identified patients as having poor understanding, poor adherence, or severe disease. Along these lines, a recent multicenter survey reported that physicians most commonly attributed readmissions to patient factors such as poor understanding or poor social support [11]. Taken together, our results and those of our predecessors suggest that clinician attention and expertise may disproportionately center on variables in the “social” realm (patients with poor understanding, adherence, or support), but may also be limited by clinician “blind spots” where further work to improve the usefulness of readmission prediction tools might be productively directed. In their report, Allaudeen et al. attempted to address these “blind spots” by assessing if clinicians could correctly predict the reasons for potential readmissions. They found that providers generally underestimated the risk of complications of therapy, and hypothesized that this may have contributed to providers’ poor ability to predict readmissions [7]. Our results could be interpreted as supporting this result: when physicians identified patients as receiving high-risk therapy, the AUC was lower (Table 4). An alternative explanation, however, might be that high-risk therapy is an overall less reliable risk factor for hospital readmission.

Our work has some limitations. First, our results were obtained in a cohort of physicians and patients in a single medical center, and may not be applicable to patients and physicians in other settings. Second, our mechanism of detecting readmissions was unable to detect patients readmitted to hospitals not covered by our hospitalist practice group. Because the LACE index and provider estimation of readmission was generally high for both readmitted and non-readmitted patients, the most likely effect this had on our results was to dilute the predictive capacity of both the providers and the LACE index, though we cannot rule out that patients who were readmitted were actually assigned a low risk of readmission by providers, and a high LACE index, which would improve the performance of the LACE index while worsening the performance of the providers. Third, the generation of the LACE index through our EHR depends on completing the problem list in the EHR, a process that is dependent on providers, and which would affect the LACE index if not done accurately. The LACE index has a possible range of scores of 1–19, whereas we asked providers to predict the risk of readmission on a scale from 0 to 100. We did this because percentages are terms that are familiar to clinicians, and to facilitate the construction of ROC curves, however the more expanded scale available to providers may have biased the results in their favor. Finally, the identification of risk factors for readmission was subjective, and may have been limited by clinicians’ lack of knowledge regarding patients’ circumstances (i.e. patients would not have been identified as being nonadherent to therapy if the clinicians were unaware of their nonadherence).

## Conclusions

We have shown that clinicians are able to predict 30-day hospital readmissions as well as an industry-standard prediction tool. However, both clinician predictions and available prediction tools remain suboptimal. The LACE index, an objective tool for predicting readmissions, added additional predictive capacity to less experienced (resident) clinicians, but its usefulness was limited in a patient population with a high burden of medical comorbidities, and it did not improve the predictions of experienced clinicians. Clinicians demonstrated relative strength in predicting readmissions in patients with poor understanding of their illness or adherence to their therapies. Further work should be directed at developing tools that enhance clinicians abilities to predict hospital readmissions.

## Abbreviations

95% CI: 95% Confidence Interval
AUC: Area under the curve
CCI: Charlson comorbidity index
EHR: Electronic health record
OR: Odds ratio
POLST: Physician orders for life-sustaining treatment
Pra: Probability of readmission score
Q1: 25th percentile
Q3: 75th percentile
ROC curve: Receiver operating characteristic curve
SD: Standard deviation
